# Supplementary material for: Integration of mathematical modeling and economics approaches to evaluate strategies for control of Salmonella Dublin in a heifer-raising operation
Source: PLoS One. 2025 Oct 16;20(10):e0332465. doi: 10.1371/journal.pone.0332465 (PMC12530588; doi:10.1371/journal.pone.0332465)
Supplement: S1 Table — The number of cattle on the farm (N) is 1,000. (DOCX) [file pone.0332465.s003.docx]

|  |  |  |  |  |  |  |  |  |
| --- | --- | --- | --- | --- | --- | --- | --- | --- |
| Animal state | Number of iterations |  |  |  |  |  |  |  |
|  | 100 |  | 500 |  | 1,000 |  | 5,000 |  |
|  | Mean | CoV^a^ | Mean | CoV | Mean | CoV | Mean | CoV |
| Susceptible | 37.8 | 0.6 | 35.6 | 0.5 | 35.7 | 0.5 | 32.8 | 0.5 |
| Infectious^b^ | 10.0 | 2.3 | 11.7 | 1.6 | 11.3 | 1.6 | 11.4 | 1.6 |
| Recovered | 52.2 | 0.4 | 52.7 | 0.4 | 53.0 | 0.35 | 52.8 | 0.35 |

^a^ CoV=Coefficient of variation

^b^Proportion of individuals in the Asymptomatic (*A*), Clinically ill (*I*), and Carrier (*C*) compartments by the end of the 2-year simulation period (initial herd size = 1,000).
