# Supplementary material for: Integration of mathematical modeling and economics approaches to evaluate strategies for control of Salmonella Dublin in a heifer-raising operation
Source: PLoS One. 2025 Oct 16;20(10):e0332465. doi: 10.1371/journal.pone.0332465 (PMC12530588; doi:10.1371/journal.pone.0332465)
Supplement: Attachment — Submitted filename: Response to Reviewers.pdf [file pone.0332465.s011.pdf]

We sincerely appreciate your comments and suggestions on our draft. Please find our detailed responses below.

Reviewer #1:

Comment: Benefit of the Study to Modeled HRO: It would be helpful to explicitly highlight how this study enhances the understanding of *S*. Dublin transmission within a single heifer-raising operation (HRO). Additionally, since the model is based on an HRO, discussing its applicability to other cattle herd types would add value.

R. Thank you very much for your comment. We included additional sentences in the "Discussion" section to indicate the contribution of our model to understanding *S.* Dublin transmission within a HRO. Specifically, we now indicate the following in the discussion section:

"Our model advances the understanding of *S*. Dublin transmission within a HRO by highlighting the important role of clinically ill cattle in herd-level spread, the reduced impact of carriers in S. Dublin dissemination past the initial pathogen introduction, and the relevance of pen environment contamination as a mechanism for infecting incoming calves" (Lines 638-642).

Regarding the generalizability of our model to other systems, we included sentences to indicate that the extent to which our model applies to other cattle operations is restricted (Lines 650-664):

"Key characteristics of most HROs in the US, such as the absence of pre-weaned and lactating cattle, limit direct application of our model or its findings to other cattle systems. Additionally, findings from our model should not be applied to operations that raise cattle in open lots or pastures, as the environmental conditions in those settings meaningfully differ from those in barns. Nonetheless, certain insights from the model, particularly the importance of environmental contamination in sustaining transmission, may still be useful for understanding *S*. Dublin dynamics in other intensively managed systems. For instance, while most feedlots differ from HROs in their open-lot design, some share management features such as the grouping of animals by age and structured cohort movement. These similarities suggest that the model's structure could be adapted to explore transmission dynamics in feedlot settings. Overall, we expect the model to be used by other researchers to predict the epidemiological and economic consequences of *S*. Dublin in HROs. As a mathematical abstraction describing *S*. Dublin spread within a herd of growing cattle operating on a batch system, general insights from our model could be of value to other cattle systems that could be similarly abstracted."

Comment: Realism of Cleaning Assumption: The assumption of cleaning a farm 12 times per day seems quite intensive. The authors could discuss the realism of this scenario and, if possible, provide references or practical justifications for this assumption.

R. We understand your concern about frequent cleaning. The intervention scenarios involving frequent cleaning and vaccination were designed not only to reflect current practices but also to explore potential approaches that are currently impractical/unfeasible and would require further technological development for their implementation. For instance, if our study findings had

supported the notion that frequent cleaning is ineffective, there would be little justification for investing in new technologies to support it. Additionally, if more effective cleaning becomes practically feasible, it would also offer additional benefits, such as reducing ammonia emissions. We updated the “Discussion” (Lines 754–758 and lines 761–762) section to highlight potential additional benefits of developing strategies for improved cleaning and vaccination. The edited text reads as follows:

“It is important to note that some of the stringent cleaning frequencies evaluated in this study, such as cleaning 6x or 12x times per day, may not be feasible in US HROs at present. However, our goal with this assessment was to determine the effectiveness of these practices if they were to be implemented in a HRO and whether developing the technology to achieve it would be a worthwhile resource investment.”

Comment: Transmission Pathways and Indirect: Transmission Assumption The study assumes that susceptible individuals become infected primarily through indirect transmission via the environment contaminated with S. Dublin bacteria. However, some studies suggest that farm-to-farm movements are the main driver of spread (e.g., https://www.sciencedirect.com/science/article/pii/S0022030224008166). Since the authors acknowledge in line 526 that HROs play a role in dissemination, and again in line 778 regarding cattle movements, it would be useful to discuss *S*. Dublin spread via movements in more detail.

R. Thank you for your comment. We agree that farm-to-farm movements, particularly through the movement of animals, can play an important role in the dissemination of *S*. Dublin between dairy operations. We now acknowledge this in the “Discussion”, specifically in the “Model limitation” section (Lines 912–921). Briefly, we now indicate that our model does not account for the transmission of *S*. Dublin between farms via animal movements, which has been found to play an important role in *S.* Dublin spread elsewhere. The paragraph reads as follows:

“Furthermore, our model focused on within-farm transmission, assuming that indirect contact through contaminated environments is the primary route of infection among cattle. As a result, the broader implications of animal movement between farms, such as the risk of introducing infection to naïve herds during transportation and triggering outbreaks, were beyond the scope of this study and therefore not assessed. We acknowledge this as a limitation of our study, since farm-to-farm transmission through cattle movement has been identified as an important mechanism for *S*. Dublin spread between dairy operations [77]. Future studies evaluating transmission between HROs and farms would provide valuable insights into the role played by these operations in the broader dissemination of *S*. Dublin in the US.”

Comment: Testing and Quarantine Strategies: Line 547 mentions that testing cattle before returning to their farms and quarantining animals upon arrival could help prevent the introduction of S. Dublin. It would be helpful to discuss how long before returning to the farm testing should occur. The authors may refer to the Danish study mentioned above (https://www.sciencedirect.com/science/article/pii/S0022030224008166) for additional context

R. Thank you very much for this comment. We incorporated the suggested article into the discussion to highlight the importance of the timing of testing to prevent the dissemination of *S.* Dublin to other operations (Lines 689–694). Nonetheless, we indicate that testing has severe limitations at the moment and improvements are needed to implement it as a surveillance strategy (Lines 694–697). The paragraph reads as follows:

"A recent Danish study [77] found that cattle movements in the month preceding detection were the strongest predictor of *S*. Dublin infection in a herd, underscoring the importance of movement-related biosecurity. However, the effectiveness of pre-movement testing depends on the availability of reliable diagnostics, which remains a major limitation. Currently available tests have low sensitivity [11,48]. When within-herd prevalence is low, detecting infected herds becomes particularly challenging due to the reduced likelihood of sampling a positive animal [78]. Thus, while pre-movement testing holds promise in principle, significant improvements in diagnostic accuracy would be necessary before it could be implemented as a reliable surveillance strategy."

Comment: Test Sensitivity and Within-Herd Prevalence: Line 551 states that the available tests have low sensitivity. The authors could briefly discuss how test sensitivity varies with within-herd prevalence, as this could impact the effectiveness of detection and control strategies

R. Thanks for the suggestion. We now include an additional sentence to indicate the particular importance of test sensitivity in scenarios with low within-herd prevalence (Lines 694–697). The updated sentences indicate the following:

"When within-herd prevalence is low, detecting infected herds becomes particularly challenging due to the reduced likelihood of sampling a positive animal [78]. Thus, while pre-movement testing holds promise in principle, significant improvements in diagnostic accuracy would be necessary before it could be implemented as a reliable surveillance strategy."

Comment: Duration of the Simulation Period: The manuscript simulates S. Dublin spread over a two-year period (lines 275 and 777). Given that the Danish study mentioned above reports a median infection duration of 25 months in farms, which aligns well with the chosen simulation period, the authors could briefly discuss how model results would be influenced by choosing a longer or shorter period

R. Thanks for your comment. We now include clarification in the methods to justify the decision to select 2-years as the simulation period (Lines 347–350):

"The 2-year simulation length was selected because *S*. Dublin dynamics show no remarkable variations after two years into the simulation, while selecting a shorter period provides a narrower understanding of *S*. Dublin epidemiological and economic impacts on a HRO."

Reviewer #2

Comment: Overall, this study presents a strong model with practical implications, but refining its focus and clarity will maximize its utility for both researchers and industry stakeholders. I believe the study currently attempts to cover too much ground in a single manuscript and would be beneficial to split it into two papers: one focusing on the within-farm model itself (per se already innovative), including sensitivity analyses of key parameters, and another dedicated to the economic evaluation of mitigation strategies. If the authors prefer to keep the structure as is, then some details need to be made clearer in the text, and the inclusion of some supplementary information in the core text of the manuscript should be reconsidered.

R. We deeply appreciate your suggestions. After much consideration, we decided to keep the article in the same format (everything together), while improving the manuscript's readability based on your recommendations. This meant bringing details from the supplementary material to the main text (including model equations), removing results from the discussion, and adding descriptions when needed to improve clarity.

Major comments:

1. Introduction - Context on S. Dublin epidemiology and pathobiology

Comment: The introduction would benefit from a clearer explanation of S. Dublin's epidemiology and how it differs from other Salmonella spp. It is important to justify why a specific model is needed for this pathogen.

R. Thank you very much for your comment. We have expanded the introduction by considering your suggestions. You can find these updates on lines 54–59 and 66–69. The need for a model specific to *S.* Dublin is indicated in lines 84–86. The new paragraphs read as follow:

"Unlike other non-typhoidal Salmonella serotypes, *S*. Dublin is associated with more severe invasive disease in humans and host-adapted to cattle, with the ability to persist within herds for extended periods [3]. The establishment of *S*. Dublin carriers within cattle herds hinders its control and eradication due to its ability to persist asymptomatically in infected cattle, periodically trigger outbreaks of disease, and evade detection due to intermittent shedding [4,5]." (Lines 54–59)

"Once introduced into dairy herds, *S*. Dublin can cause clinical disease in calves and abortion in cows and poses a risk to human health through the potential for bloodstream infections following the consumption of contaminated animal products such as undercooked beef or raw milk [3,10]." (Lines 66–69)

"The ability of *S*. Dublin to cause severe systemic disease in young cattle, its widespread MDR, and its ability to persist long-term undetected within herds warrant its assessment separately from other *Salmonella* serovars" (Lines 84–86)

Comment: A more detailed discussion of transmission dynamics, including the role environmental contamination (e.g., feces versus other sources) is not sufficiently discussed. This information is crucial for understanding why transmission dynamics were central to informing the model structure.

R. We appreciate this suggestion. We added a short paragraph in the introduction incorporating your suggestions on the importance of environmental contamination in *S.* Dublin transmission. This new paragraph can be found in lines 73–83:

"The primary route of *S*. Dublin infection in cattle is oral ingestion of the bacterium, with fecal contamination serving as a key contributor to the contamination of feed, water, pen surfaces, bedding, and equipment [11]. In addition to feces, *S*. Dublin is shed in bodily fluids such as milk, saliva, and nasal secretions [5]. Asymptomatic carriers can intermittently shed the pathogen, leading to ongoing environmental contamination and continued exposure of susceptible animals to infectious sources [2,5]. Notably, *S.* Dublin can survive for extended periods in the environment, including in bovine feces slurry, where it has been reported to persist for months [12], enabling sustained transmission even in the absence of clinically ill animals. Because the contaminated environment plays a key role in *S*. Dublin transmission and persistence within cattle operations, incorporating environmental factors is critical for accurately characterizing its epidemiology and informing effective control strategies."

Materials and methods - Epidemiological module

Comment: 2.1. The model assumes a single asymptomatic calf introduces infection; however, in reality, multiple infected animals can contribute to the spread. While this assumption is addressed, according to the supplementary materials, it should be made explicit in the text whether these scenarios were tested. Every reader will likely question this point, and I recommend these results to be clearly reported.

R. We agree with this comment, and we added the importance of multiple introductions explicitly in the methods (brought to the main manuscript from the Supplementary appendix) and the results sections. These changes are found in lines 467–474 and 599–605. Consequently, this information is no longer mentioned in the Supplementary Appendix. The complete paragraphs read as follows:

New paragraph in "Methods" section:

"We also assessed seasonal variation (e.g., temperature effects), herd sizes, and reductions in shedding from asymptomatic and carrier animals compared to clinically ill. Additional scenarios explored the presence or absence of shedding across infectious states, the effectiveness of cleaning at each instance, and the introduction of different numbers of asymptomatic and/or carrier calves at the start of the simulation (t = 0). The importance of uncertainty in the transmission rate, the probability of carrier development in the asymptomatic compartment, and the probability of carrier development among clinically ill animals was addressed by assessing different values for these parameters (Table S2)." (Lines 467–474)

New paragraph in the “Results” section:

“Findings from the scenario analysis also indicate that introducing multiple infectious index cases into the HRO at time t=0 did not notably increase the risk and consequences of *S*. Dublin infection compared to a single index case introduction. After the introduction of a single or multiple asymptomatic or carrier individuals at time t=0, the likelihood of a *S*. Dublin outbreak occurring when no mitigation strategies were in place was almost identical to the baseline scenario (Table S3). Likewise, only very minor differences were observed in other epidemiological outcomes (Table S3).” (Lines 599–605)

Comment: 2.2. Direct contact between heifers is not explicitly modeled, though it could significantly influence transmission dynamics. Consider incorporating this to enhance realism. Moreover, it is also not clear whether you have considered spatial heterogeneity in the model (animals within-stages might be in closer contact compared to animals between-stages).

R. Thanks for the comment. In our model, we consider the fecal-oral route as the primary pathway for transmission. We believe that the main route of direct transmission would occur if a susceptible animal ingests bacteria directly from fecal material present at the recto-anal region of an infected animal, which is the body area most likely to be contaminated by its own feces). In contrast, other parts of the animal’s body are more likely to become contaminated through contact with the environment, which can be contaminated by any infectious animal in the pen. Therefore, ingestion of bacteria from contaminated hides, other than the recto-anal region, is modeled as indirect transmission mediated by the shared environment, rather than direct contact between animals (Velasquez-Munoz et al. 2024; https://doi.org/10.3389/fvets.2023.1331767). The direct respiratory transmission of S. Dublin is considered possible but uncommon, and because its role remains insufficiently studied, it was not included in our model. We now indicate this in lines 147–154 as follows:

“Direct transmission was considered comparatively much less important in the spread of S. Dublin and was thus ignored. That is because direct transmission mainly occurs when a susceptible animal ingests fecal material from the recto-anal region of an infectious individual, while contamination of other body areas is considered indirect and mediated by the environment. The direct respiratory transmission of *S*. Dublin is considered possible but uncommon [5], and because its role remains insufficiently studied, it was not included in our model. We therefore consider fecal-oral transmission via the shared environment as the exclusive transmission route in our model [10].”

Regarding spatial heterogeneity, we now clearly specify that our model incorporates spatial heterogeneity through division of animals into distinct pens and with different probability of infection spread within vs across pens. This is now indicated in lines 212–217 as follows:

“Accordingly, the environmental compartment E was divided into twelve distinct sections (E_n), where the subscript n corresponds to one of the twelve raising stages in an HRO. Each section represents the immediate environment specific to its corresponding raising stage (pen). In this way, the model incorporates spatial structure by dividing animals into distinct pens, allowing for

a higher probability of infection spread within pens than between them, with *S*. Dublin spreading across pens at a slower rate (κ).”

Comment: 2.3. The model considers asymptomatic, clinical and carrier states, but does not explicitly account for individual variability in shedding patterns (e.g., “super-shedders” or intermittent shedding in carrier states). Have you considered incorporating a probabilistic approach for shedding rates instead of a simple fold reduction? For instance, a gamma distribution or a two-state Markov process could better reflect this variability. Particularly for carrier states, there are periods with inactivity in shedding, and these I believe should be accounted for.

R. Thank you for this insightful comment. We did not include intermittent shedding due to a lack of data on how frequent individuals resume and stop shedding. While incorporating a probabilistic approach could better reflect variability in shedding, it would require additional parameters that cannot be supported with available data and would thus require assumptions. Also, we believe that intermittent shedding is likely to have less impact when several animals are infected at the same time, unless their shedding and non-shedding episodes happen to be synchronized. Since such synchronization is unlikely, the overall contribution to environmental contamination would resemble a continuous flow, even if individual animals shed intermittently. These reasons led us to choose a more parsimonious approach. We indicate this reasoning in the limitations section now in lines 868–876 as follows:

“We chose not to incorporate intermittent shedding into the model because there is limited empirical data on how often and for how long carriers stop and resume shedding. Accounting for this variability would require introducing additional assumptions and parameters that are not well supported by current evidence. Moreover, when multiple animals are infected at the same time, intermittent shedding is unlikely to affect overall environmental contamination unless their shedding patterns are highly synchronized, which is improbable. Given these considerations, we opted for a simpler approach that assumes continuous shedding during the carrier state.”

3. Materials and methods – Infection control strategies

Comment: 3.1. The assumption that vaccination only reduces mortality in clinically ill calves may underestimate its benefits and lead to biased estimates of the number of infected and carrier animals leaving the HROs. Vaccines often reduce clinically severity but can also impact shedding levels and reduce susceptibility in healthy animals in some cases. While it is unclear, EnterVene-d (commonly used in North-American dairies), may reduce shedding levels, though only proprietary is reported. While the authors acknowledge the lack of data on shedding reduction, sensitivity analysis should be applied to better capture uncertainty in vaccines’ role in controlling S. Dublin. Was this performed?

R. Thank you very much for this comment. We added additional scenarios to assess the impacts of 25%, 50%, and 75% reduction in (i) susceptibility to *S*. Dublin, (ii) length of the

infectious period, and (iii) shedding rate (Lines 456–466) as additional vaccine effects. These are the new sentences:

“In addition to the reduction in the probability of death through vaccination, we also considered hypothetical vaccine effects that have not yet been demonstrated for existing commercial *S.* Dublin vaccines but could be valuable to assess to inform future vaccine improvements. These include 25%, 50%, and 75% VE in reducing (i) susceptibility to *S.* Dublin, (ii) length of the infectious period, and (iii) shedding, as follows: $\beta * (1 - VE)$ for reductions in susceptibility, $D_{A/I} * (1 - VE)$ for reductions in the infectious period, and $\varepsilon_n * (1 - VE)$ for shedding reduction in clinically ill and $\lambda_n * (1 - VE)$ for shedding reductions in asymptomatic and carriers; where the subscript “n” represents a specific raising stage. For these hypothetical vaccination scenarios, we only estimated their effectiveness in deaths, abortions, and asymptomatic and carrier departures, but we did not include them in the economic analysis.

Results for these hypothetical vaccination scenarios are presented in Lines (538–551) as follows:

“Findings from the scenario analysis of hypothetical vaccines showed that susceptibility reduction and shedding reduction produced identical epidemiological outcomes, as both acted on complementary components of transmission dynamics in our model equations (Equations 1, 2, 3, and 6). For these scenarios, small vaccine-driven reductions of 25% in susceptibility and shedding led to approximately 20% fewer deaths (median = 19; IQR = 8–35) compared to a “no vaccination” scenario by the end of the first year. Furthermore, carrier replacement heifers decreased by about 7% (median = 14; IQR = 8–18) with a 25% reduction in susceptibility and by up to 67% (median = 5; IQR = 1–12) with a 75% reduction by the end of the first year due to the lower number of infectious animals present at the same time in the HRO. Shortening the infectious period produced the largest impact on asymptomatic replacements, with reductions of up to 72% (median = 16; IQR = 9–23). The median number of abortions declined by about 10% (median = 9; IQR = 5–14) at a 25% reduction in susceptibility and shedding (Table S3). Impacts of these hypothetical vaccine effects were similar between the first and second year.”

And briefly discussed in Lines 720–724:

“Nonetheless, our assessment of additional hypothetical vaccination scenarios indicates that even vaccines with low effectiveness (25% reduction) in reducing susceptibility to *S*. Dublin, shortening the infectious period, and decreasing shedding can help lower *S*. Dublin-related mortality and abortions in HROs. These findings strongly suggest that such effects should be considered relevant during vaccine development and clinical trials.”

Comment: 3.2. I think cleaning frequency assumptions and the practical feasibility of scenarios should be reconsidered. While increasing cleaning frequency appears to reduce transmission, the assumption of near-linear improvement may not be realistic. The model uses $-\ln(1-Cl_{freq})$ to estimate cleaning effectiveness, which suggests diminishing returns with higher frequencies. However, the assumed 95% removal per event does not account for real-world variability in farm practices or recontamination rates. Additionally, is there biological justification for detecting a

meaningful difference between cleaning 6x and 8x per day? Would it be more appropriate to model cleaning efficacy rather than frequency to better reflect practical constraints?

R. We appreciate this comment and would like to clarify that our model does not assume near-linear improvement with increased cleaning frequency. We made the following adjustments to the manuscript:

"Equation 8 is based on the exponential distribution formula used for converting the probability of an event into the rate of the event (which is needed to model the event in an ODE model). While probability is bounded by 1, the corresponding rate is unbounded; thus, as Cl_{freq} approaches 1, μ increases rapidly, approaching infinity. It should be noted that Cl_{freq} is a function of both the cleaning effectiveness per cleaning instance (*H*) and cleaning frequency. Similar Cl_{freq} can be achieved with different combinations of the two parameters (e.g., a low H can be compensated by higher cleaning frequency and the opposite). Importantly, increases in cleaning frequency lead to larger gains in Cl_{freq} at lower frequencies, while improvements become smaller as the frequency increases reflecting diminishing returns." (Lines 327–336)

We focused on cleaning frequency because it mechanistically represents the ongoing accumulation of fecal material over time. Even with high efficacy during each cleaning event (*H*), infrequent cleaning allows contamination to build up between events and reduces overall effectiveness. Our sensitivity analysis also indicated that changes in cleaning efficacy per event (*H*) had little influence on the model's outcomes compared to changes in cleaning frequency (Table S3).

4. Discussion

Comment: There is some repetition of results in the discussion. Streamlining key points and ensuring that all data are fully presented in the results section before being discussed would improve conciseness.

Moreover, some claims need stronger support from results. For example, while the study highlights the role of HROs as disseminators of S. Dublin, this is a within-farm model, so explicit results demonstrating spread beyond the HRO are necessary for this claim.

R. Thank you for your feedback. We have revised the discussion to avoid repetition of results in the discussion section. You can see in the tracked-changes version of the document that we have removed some paragraphs with redundant information in the Discussion section (e.g., lines 668–675 and 772–775)

Regarding the role of HROs in *S.* Dublin dissemination to other facilities, we have clarified across the manuscript that our findings imply a potential contribution rather than directly demonstrating spread beyond the HRO (e.g., lines 939–940).

<u>Minor comments:</u>

Comment: [lines 56-58] – some references describe multi-drug resistance in Humans; emphasize the relevance of this study for public health in the introduction as well.

R. Thanks for your comment. We added a sentence in the manuscript incorporating your suggestion (lines 66–69). The sentence reads as follows:

"Once introduced into dairy herds, *S*. Dublin can cause clinical disease in calves and abortion in cows and poses a risk to human health through the potential for bloodstream infections following the consumption of contaminated animal products such as undercooked beef or raw milk [3,10]."

Comment: [line 63] – "most effective" to "most cost-effective"

R. We made the suggested amendment (Line 71).

Comment: [lines 63-64] – "i.e., yielding the best outcome in a real-life scenario" is unclear and unnecessary, can be removed or should be replaced

R. We made the suggested amendment.

Comment: [lines 82-83] – emphasize that an economic approach such as this one, to evaluate cost-effective mitigations strategies, has not been used in the US.

R. Thanks for your comment. This suggestion is now reflected in the manuscript's lines 104–106.

Comment: [line 85] – "mathematical model" – specify the type of model stochastic, mechanistic, compartmental, within-farm?

R. We now indicate "mechanistic compartmental" mathematical model (line 108)

Comment: [line 86] – "evaluate the vaccine effectiveness and cleaning improvements" to "evaluate the effectiveness of vaccination and different cleaning strategies for controlling S. Dublin infection"

R. Thanks for the suggestion. You can now see it incorporated in lines 109–110.

Comment: [line 96] – 53.9% of HROs seems relatively low? Need a little more context on what differentiates these HROs with the remaining 46.1%. And why 2011? I believe there is a more census but please confirm.

R. Thank you for this observation. We have clarified in the text what the remaining 46.1% of operations represent (lines 120–121). While 53.9% may seem modest, it reflects more than half of HROs in the US. We confirm that the 2011 USDA APHIS report is the most recent and comprehensive source of national data available for characterizing HROs in the US.

Comment: [line 102 – Figure 1] – Do calves only arrive from dairy farms? If this if not clear, I would remove the word “farms” and replace by something like “facilities”, since they could arrive from comingling locations such as livestock markets, sorting facilities and other calf dealers, so it avoids an oversimplistic description of US cattle production and distribution chains.

R. We incorporated this change into the Figure legend as suggested. Thank you.

Comment: [lines 106-109] – What characterizes differences between R2-R8 and R9-12? Why 45 days interval? Would help the reader to be a little clearer.

R. Thank you for this comment. We have revised the text to clarify that the distinction between raising stages R2–R8 and R9–R12 relates to differences in animal physiological status and model outcomes: individuals in R9–R12 are considered pregnant and may experience abortion, while those in R2–R8 are not pregnant and can die from infection. These stages also differ in feces production, which affects environmental contamination. The 45-day interval was chosen to reflect the arrival of heifers in batches and to allow for more precise estimation of age-specific costs, including losses due to death or abortion within each stage. We have updated the text accordingly to improve clarity (Lines 130–136):

“During this period, individuals progress through twelve raising stages: one as a weaned calf (R1; 90–135 days old), seven as growing heifers (R2 to R8; 136–450 days old), and four as pregnant heifers (R9 to R12; 451–630 days old). In contrast to other raising stages, pregnant heifers (R9 to R12) cannot die from infection, but they may experience abortion. Individuals transition between stages every 45 days to reflect the arrival of heifers into the HRO in batches and to allow for more precise estimation of age-specific costs, including losses due to death or abortion within each stage.”

Comment: [line 107] – Growing heifers from R2-R8 and pregnant heifers from R9-R12? R8 seems repeated.

R. Thanks for pointing that out. Now we properly separate each category as you indicated.

Comment: [lines 136-137] – I believe it would make more sense to include decreased susceptibility at each infection cycle since this is reported in the literature or look at it during sensitivity analysis.

R. Thank you for the suggestion. We did not include decreased susceptibility in the model because doing so would have required duplicating or triplicating each compartment to represent individuals with varying levels of resistance. This would have significantly increased the model's complexity and computational demands. Additionally, it would require additional parameters to describe the reduced susceptibility following one (or more) past infections, which are currently unknown. Nevertheless, this simplification is expected to impact model predictions. Thus, we acknowledged this as a major limitation of the model and discussed the direction of expected bias in model predictions due to this simplification. This is indicated in Lines 897–902:

"The model did not account for increased resistance to *S*. Dublin infection among susceptible individuals who had previously been infected and returned to the susceptible compartment upon the loss of immunity [89]. This exclusion was due to the limited data for parameterization. The inclusion of this feature in the model could have reduced the incidence of *S*. Dublin reinfections in previously exposed cattle and ultimately decreased health impacts. However, our findings are relevant to a naive herd and thus represent conservative predictions."

Comment: [lines 220] – Are the studied vaccines specific for S. Dublin? I would report either here or in the limitations since studies in the literature report genomic differences that might lead to vaccines being less effective.

R. Thank you for this important point. In our model, we only assessed EnterVene-D, a vaccine developed specifically for *S*. Dublin, and we have now added some scenarios exploring vaccine efficacy when reducing shedding and duration of infectiousness. The vaccines shown in Figure 3 target *Salmonella* spp. and some of them target *S*. Dublin. We have clarified this in the legend, which now reads: "Fig 3. Experimental and commercial vaccine effects against *Salmonella* spp. and *S.* Dublin infection in cattle assessed and reported in the literature." (Lines 284–295)

To further address your concern, we have added a sentence in the Methods section, following the mention of Figure 3, noting that genomic and antigenic variability among *Salmonella* serovars may influence vaccine efficacy, as most vaccines are developed from specific laboratory strains that may not represent the diversity found in the field (lines 270–273). The sentence reads as follows:

"It is important to note that, despite the variety of vaccine effects reported in the literature, genomic and antigenic heterogeneity among *Salmonella* serovars may influence vaccine effectiveness, as most vaccines are developed using specific laboratory strains that may not represent the full diversity present in the field [57, 58]."

Comment: [lines 333-344] – I suggest mentioning here how many and what specific scenarios of cleaning + vaccination (individually and combined) were included. This would make the results more clear.

R. We appreciate your comment. We expanded the “Scenario analysis” subsection (lines 454–456) to clearly indicate what specific scenarios were assessed in our study. The sentence reads as follows:

“These include eight cleaning frequency scenarios (cleaning 3x/week to cleaning 12x/day), one vaccination scenario (commercial vaccine), and eight scenarios assessing the combination of cleaning frequencies and vaccination”

Comment: [lines 486 - 488] – These results emphasize that we need to be careful when defining shedding rates in our model. Intermittent shedding is likely in carriers and might limit the results (?)

R. Thank you for this important observation. We have clarified in the “Identified knowledge gaps in *S*. Dublin epidemiology” subsection of the “Discussion” that our results are based on a model that does not account for intermittent shedding and explained why we believe this assumption does not substantially impact our findings. New sentences in lines 868–876 indicate the following:

“We chose not to incorporate intermittent shedding into the model because there is limited empirical data on how often and for how long carriers stop and resume shedding. Accounting for this variability would require introducing additional assumptions and parameters that are not well supported by current evidence. Moreover, when multiple animals are infected at the same time, intermittent shedding is unlikely to affect overall environmental contamination unless their shedding patterns are highly synchronized, which is improbable. Given these considerations, we opted for a simpler approach that assumes continuous shedding during the carrier state.”

Comment: [line 500-501] – “(i) HROs play an important role in the spread…” and [lines 515-516] – “identified the role that these operations might be playing as disseminators of the infection to dairy herds” - what results show this? I think you need to report this better in the results, but since this is a within-farm model I think the role of HROs as disseminators of infection to dairy herds is not a main result of the study.

R. Thank you for your comment. We have revised the text to remove the statement that “HROs play an important role in the spread…” as a direct finding. Instead, we now present it as an implication of our results as follows (627–629):

“Our findings also imply that HROs play a meaningful role in the transmission of S. Dublin to dairy cattle herds. The implications of these results, along with the study’s limitations, are discussed in the following sections.”

Comment: [line 501] – “(ii) frequent cleaning (…)” what do you mean by frequent? Which scenarios were more effective? Needs to be more specific

R. Thanks for the comment. We provide more details when presenting the information now (lines 619–620) as follows:

“The main study findings are: (i) cleaning multiple times per day, with cleaning 12x/day being the most effective…”

Comment: [lines 522-524] – How are stakeholders expected to use this model? Is there a shiny app? Otherwise, I recommend replacing by other researchers.

R. Thanks for this comment. We have made the recommended change (Lines 660–664) as follows:

“Overall, we expect the model to be used by other researchers to predict the epidemiological and economic consequences of *S*. Dublin in HROs. As a mathematical abstraction describing *S*. Dublin spread within a herd of growing cattle operating on a batch system, general insights from our model could be of value to other cattle systems that could be similarly abstracted.”

Comment: [lines 528-533] – Are the estimates of number of departing asymptomatic and carrier replacement heifers presented in the results section? I could not find it

R. Yes, this is presented in lines 522–523 and 528–529.

Comment: [line 620-621] – “introduction of an infectious individual” – asymptomatic infectious, correct?

R. Yes, this is correct. Nonetheless, changes recommended by reviewers led to this particular sentence being removed.

<u>Additional Considerations:</u>

Comment: Parameter Distributions: It is not clear why beta distributions (Beta-PERT) were chosen for specific parameters (e.g., vaccine efficacy). Clarifying these choices in the methods section would strengthen transparency.

R. Thanks for your comment. We now indicate why we chose Beta-PERT in the footnote of Table 1 since we do not describe specific distributions in the main text. The reasoning is that the Beta-PERT distribution is useful when available data about a particular parameter is limited, but enough to provide a minimum, most likely, and maximum value.

Comment: Transmission Assumption: The model appears to use a density-dependent transmission term via environmental contamination, according to the code. Explicitly stating that

the model is density-dependent (rather than frequency-dependent) and its justification in the methods section would be helpful.

R. Yes, that's correct. This is now explicitly indicated in the Methods (lines 143–147):

"This model considers a density-dependent transmission process, where the risk of infection increases with the density of bacteria in the environment, which increases with cattle density on the farm. We chose a density-dependent formulation to reflect the biological assumption that in a denser population, cattle are more likely to come into indirect contact through contaminated environment and spread the infection."

Comment: Herd Size: Results for different herd sizes (500 vs. 2000 heifers) appear in supplementary tables (S2, S3) but are not discussed in the main results section. If relevant, these should be reported.

R. We appreciate this suggestion. To keep the manuscript at a reasonable length, we decided to keep these results in the Appendix.

Comment: Seasonality: same comment as above.

R. Like the previous response, we decided to keep seasonality in the Appendix.

Comment: Table S2: Caption is missing, or at least a full description should be included for PoO, DACA and OI. It is also not clear to me what the X's represent.

R. Thank you for pointing this out. An updated caption is available at the end of the reference section based on journal requirements indicating the following (Lines 1,251–1,252):

"Cells marked with an 'X' indicate that the corresponding outcome was estimated in that scenario." This information was also added as a footnote in Table S2.
