# Supplementary material for: Integration of mathematical modeling and economics approaches to evaluate strategies for control of Salmonella Dublin in a heifer-raising operation
Source: PLoS One. 2025 Oct 16;20(10):e0332465. doi: 10.1371/journal.pone.0332465 (PMC12530588; doi:10.1371/journal.pone.0332465)
Supplement: Attachment — Submitted filename: Review.docx [file pone.0332465.s009.docx]

Dear Authors, thank you for the opportunity to review your manuscript. Overall, the paper is well written, and the mathematical model is clear and easy to follow from epidemiological and economic point of view. I appreciate the depth of analysis and the valuable insights provided. Below are a few comments that may help strengthen the study:

**Benefit of the Study to Modeled HRO**

- It would be helpful to explicitly highlight how this study enhances the understanding of **S. Dublin transmission using only one heifer-raising operation (HRO)**.
- Additionally, since the model is based on an HRO, discussing **its applicability to other cattle herd types** would add value.

**Realism of Cleaning Assumption**

- The assumption of **cleaning a farm 12 times per day** seems quite intensive. The authors could discuss the realism of this scenario and, if possible, provide **references or practical justifications** for this assumption.

**Transmission Pathways and Indirect Transmission Assumption**

- The study assumes that **susceptible individuals become infected primarily through indirect transmission via the environment** contaminated with S. Dublin bacteria. However, some studies suggest that **farm-to-farm movements** are the main driver of spread (e.g., <https://www.sciencedirect.com/science/article/pii/S0022030224008166>).
- Since the authors acknowledge in **line 526** that HROs play a role in dissemination, and again in **line 778** regarding the movement of cattle, it would be useful to discuss spread of S. Dublin via movements in detail.

**Testing and Quarantine Strategies**

- **Line 547** mentions that **testing cattle before returning to their farms** and **quarantining animals upon arrival** could help prevent the introduction of S. Dublin.
- It would be helpful to discuss **how long before returning to the farm testing should occur**. The authors may refer to the Danish study mentioned above ([ihttps://www.sciencedirect.com/science/article/pii/S0022030224008166](https://www.sciencedirect.com/science/article/pii/S0022030224008166)) for additional context.

**Test Sensitivity and Within-Herd Prevalence**

- **Line 551** states that the **available tests have low sensitivity**. The authors could briefly discuss how **test sensitivity varies with within-herd prevalence**, as this could impact the effectiveness of detection and control strategies.

**Duration of the Simulation Period**

- The manuscript simulates **S. Dublin spread over a two-year period** (lines **275 and 777**).
- Given that **study already mentioned above that** reports a **median infection duration of 25 months in farms**, which aligns well with the chosen simulation period, the authors could briefly discuss how model results would be influence by choosing a longer or shorter period.

I appreciate the effort that went into this study and believe these additions will further enhance its clarity and impact. I look forward to seeing the revised version.
