# Supplementary material for: Integration of mathematical modeling and economics approaches to evaluate strategies for control of Salmonella Dublin in a heifer-raising operation
Source: PLoS One. 2025 Oct 16;20(10):e0332465. doi: 10.1371/journal.pone.0332465 (PMC12530588; doi:10.1371/journal.pone.0332465)
Supplement: Attachment — Submitted filename: Review_PONE.docx [file pone.0332465.s010.docx]

**Manuscript PONE-D-25-05247**

Title: “Integration of mathematical modeling and economics approaches to evaluate strategies for control of Salmonella Dublin in a heifer-raising operation”

**Summary and overall impression**

This manuscript presents a stochastic simulation model to assess the impact of Salmonella Dublin infection and evaluate the cost-effectiveness of mitigation strategies in heifer-raising operations (HROs). The integration of an economic analysis with a modified Susceptible-Infected-Recovered-Susceptible (SIRS) model is a major strength, as it provides a novel and practical approach to understanding S. Dublin transmission dynamics and control measures. Given the increasing concern surrounding S. Dublin in dairy production and its public health implications, this study offers valuable insights for stakeholders, including farmers, policymakers, and researchers. The inclusion of model code in a public repository (GitHub) further enhances the study's transparency and reproducibility, which is commendable.

Overall, this study presents a strong model with practical implications, but refining its focus and clarity will maximize its utility for both researchers and industry stakeholders. I believe the study currently attempts to cover too much ground in a single manuscript and would be beneficial to split it into two papers: one focusing on the within-farm model itself (*per se* already innovative), including sensitivity analyses of key parameters, and another dedicated to the economic evaluation of mitigation strategies. If the authors prefer to keep the structure as is, then some details need to be made clearer in the text, and the inclusion of some supplementary information in the core text of the manuscript should be reconsidered.

As such, I recommend major revisions before further acceptance.

**Discussion of specific areas for improvement**

Major comments:

1. **Introduction -** Context on S. Dublin epidemiology and pathobiology

The introduction would benefit from a clearer explanation of S. Dublin’s epidemiology and how it differs from other Salmonella spp. It is important to justify why a specific model is needed for this pathogen. A more detailed discussion of transmission dynamics, including the role environmental contamination (e.g., feces versus other sources) is not sufficiently discussed. This information is crucial for understanding why transmission dynamics were central to informing the model structure.

2. **Materials and methods** - Epidemiological module

2.1. The model assumes a single asymptomatic calf introduces infection; however, in reality, multiple infected animals can contribute to the spread. While this assumption is addressed , according to the supplementary materials, it should be made explicit in the text whether these scenarios were tested. Every reader will likely question this point, and I recommend these results to be clearly reported.

2.2. Direct contact between heifers is not explicitly modeled, though it could significantly influence transmission dynamics. Consider incorporating this to enhance realism. Moreover, it is also not clear whether you have considered spatial heterogeneity in the model (animals within-stages might be in closer contact compared to animals between-stages).

2.3. The model considers asymptomatic, clinical and carrier states, but does not explicitly account for individual variability in shedding patterns (e.g., “super-shedders” or intermittent shedding in carrier states). Have you considered incorporating a probabilistic approach for shedding rates instead of a simple fold reduction? For instance, a gamma distribution or a two-state Markov process could better reflect this variability. Particularly for carrier states, there are periods with inactivity in shedding, and these I believe should be accounted for.

3. **Materials and methods** – Infection control strategies

3.1. The assumption that vaccination only reduces mortality in clinically ill calves may underestimate its benefits and lead to biased estimates of the number of infecteds and carrier animals leaving the HROs. Vaccines often reduce clinically severity but can also impact shedding levels and reduce susceptibility in healthy animals in some cases. While it is unclear, EnterVene-d (commonly used in North-American dairies), may reduce shedding levels, though only proprietary is reported. While the authors acknowledge the lack of data on shedding reduction, sensitivity analysis should be applied to better capture uncertainty in vaccines’ role in controlling S. Dublin. Was this performed?

3.2. I think cleaning frequency assumptions and the practical feasibility of scenarios should be reconsidered. While increasing cleaning frequency appears to reduce transmission, the assumption of near-linear improvement may not be realistic. The model uses −ln(1−Cl_freq_) to estimate cleaning effectiveness, which suggests diminishing returns with higher frequencies. However, the assumed 95% removal per event does not account for real-world variability in farm practices or recontamination rates. Additionally, is there biological justification for detecting a meaningful difference between cleaning 6x and 8x per day? Would it be more appropriate to model cleaning efficacy rather than frequency to better reflect practical constraints?

4. **Discussion**

There is some repetition of results in the discussion. Streamlining key points and ensuring that all data are fully presented in the results section before being discussed would improve conciseness. Moreover, some claims need stronger support from results. For example, while the study highlights the role of HROs as disseminators of S. Dublin, this is a within-farm model, so explicit results demonstrating spread beyond the HRO are necessary for this claim.

Minor comments:

- **[lines 56-58]** – some references describe multi-drug resistance in Humans; emphasize the relevance of this study for public health in the introduction as well.
- **[line 63]** – “most effective” to “most cost-effective”
- **[lines 63-64]** – “i.e., yielding the best outcome in a real-life scenario” is unclear and unnecessary, can be removed or should be replaced
- **[lines 82-83]** – emphasize that an economic approach such as this one, to evaluate cost-effective mitigations strategies, has not been used in the US.
- **[line 85]** – “mathematical model” – specify the type of model stochastic, mechanistic, compartmental, within-farm?
- **[line 86]** – “evaluate the vaccine effectiveness and cleaning improvements” to “evaluate the effectiveness of vaccination and different cleaning strategies for controlling S. Dublin infection”
- **[line 96]** – 53.9% of HROs seems relatively low? Need a little more context on what differentiates these HROs with the remaining 46.1%. And why 2011? I believe there is a more census but please confirm.
- **[line 102 – Figure 1]** – Do calves only arrive from dairy farms? If this if not clear, I would remove the word “farms” and replace by something like “facilities”, since they could arrive from comingling locations such as livestock markets, sorting facilities and other calf dealers, so it avoids an oversimplistic description of US cattle production and distribution chains.
- **[lines 106-109]** – What characterizes differences between R2-R8 and R9-12? Why 45 days interval? Would help the reader to be a little clearer.
- **[line 107]** – Growing heifers from R2-R8 and pregnant heifers from R9-R12? R8 seems repeated.
- **[lines 136-137]** – I believe it would make more sense to include decreased susceptibility at each infection cycle since this is reported in the literature or look at it during sensitivity analysis.
- **[lines 220]** – Are the studied vaccines specific for S. Dublin? I would report either here or in the limitations since studies in the literature report genomic differences that might lead to vaccines being less effective.
- **[lines 333-344]** – I suggest mentioning here how many and what specific scenarios of cleaning + vaccination (individually and combined) were included. This would make the results more clear.
- **[lines 486 - 488]** – These results emphasize that we need to be careful when defining shedding rates in our model. Intermittent shedding is likely in carriers and might limit the results (?)
- **[line 500-501]** – “(i) HROs play an important role in the spread…” and [lines 515-516] – “identified the role that these operations might be playing as disseminators of the infection to dairy herds” - what results show this? I think you need to report this better in the results, but since this is a within-farm model I think the role of HROs as disseminators of infection to dairy herds is not a main result of the study.
- **[line 501]** – “(ii) frequent cleaning (…)” what do you mean by frequent? Which scenarios were more effective? Needs to be more specific
- **[lines 522-524]** – How are stakeholders expected to use this model? Is there a shiny app? Otherwise, I recommend replacing by other researchers.
- **[lines 528-533]** – Are the estimates of number of departing asymptomatic and carrier replacement heifers presented in the results section? I could not find it
- **[line 620-621]** – “introduction of an infectious individual” – asymptomatic infectious, correct?

Additional Considerations

- **Parameter Distributions**: It is not clear why beta distributions (Beta-PERT) were chosen for specific parameters (e.g., vaccine efficacy). Clarifying these choices in the methods section would strengthen transparency.
- **Transmission Assumption**: The model appears to use a density-dependent transmission term via environmental contamination, according to the code. Explicitly stating that the model is density-dependent (rather than frequency-dependent) and its justification in the methods section would be helpful.
- **Herd Size**: Results for different herd sizes (500 vs. 2000 heifers) appear in supplementary tables (S2, S3) but are not discussed in the main results section. If relevant, these should be reported.
- **Seasonality**: same comment as above.
- **Table S2:** Caption is missing, or at least a full description should be included for PoO, DACA and OI. It is also not clear to me what the X’s represent.
